# Proton pump inhibitor use and risk of depression among Sudanese adults: a cross-sectional community-based study

**DOI:** 10.3389/fpsyt.2025.1689599

**Published:** 2026-01-02

**Authors:** Afnan A. Alwabili, Saeed M. Omar, Eman A. Alotaibi, Ishag Adam

**Affiliations:** 1 Department of Psychiatry, College of Medicine, Qassim University, Buraydah, Saudi Arabia; 2 Department of Medicine, Faculty of Medicine and Health Sciences at the University of Gadarif, Gadarif, Sudan; 3 Department of Family and Community Medicine, College of Medicine, Qassim University, Buraydah, Saudi Arabia; 4 Department of Obstetrics and Gynecology, College of Medicine, Qassim University, Buraydah, Saudi Arabia

**Keywords:** adult, age, depression, patient health questionnaire-9, proton pump inhibitors

## Abstract

**Background:**

Depression is a major global public health issue. There is little available data regarding the association between the use of proton pump inhibitors (PPIs) and depression. The current study aimed to investigate the association between PPIs and depression among adults in Central Sudan.

**Methods:**

A community-based cross-sectional study was conducted to examine adults in Central Sudan. Sociodemographic data and medical history were collected. Depression was assessed using the Patient Health Questionnaire-9 (PHQ-9). Multivariate binary regression analyses, modified Poisson regression analyses, and propensity score matching (PSM) were performed.

**Results:**

We enrolled 914 adults (317 [34.7%] men and 597 [65.3%] women). Participants’ median (interquartile range) age was 38.0 (27.0–52.0) years. Of the participants, 255 (27.9%) had used PPIs. In Poisson regression, after adjusting for confounders (age, sex, education, occupation, marital status, hypertension, diabetes mellitus, body mass index, smoking, alcohol), use of PPIs [adjusted relative risk = 1.30, 95% confidence interval (CI) = 1.21–1.40] was associated with a higher depression score. Of 914 adults, 155 (17.0%) had depression (PHQ-9 score of ≥ 8). Before matching (PSM), in multivariate binary regression (after removing confounders), PPIs were associated with depression [adjusted odds ratio (AOR)= 1.64; 95% CI = 1.14–2.37]. After matching, PPIs were associated with depression (AOR = 1.66; 95% CI = 1.13–2.41).

**Conclusion:**

This study revealed a positive association between PPI use and depression. Longitudinal studies will be necessary to examine this relationship in more depth.

## 1 Introduction

Depression is a major public health issue affecting millions of people globally (1). Several sociodemographic factors, including age (2, 3), marital status (2), education (2), smoking (4), body mass index (BMI) (2), and presence of comorbidity (e.g., diabetes mellitus and hypertension) (2, 4) are associated with depression among adults. Although the precise etiology of depression has not yet been established, the condition is considered to have a complex and multifactorial etiology involving a range of factors, including genetic predispositions, psychosocial stressors, neurobiological imbalances, and lifestyle (5, 6).

Proton pump inhibitors (PPIs) are the primary therapeutic drugs for treating gastric acid-related diseases, including peptic ulcers and gastroesophageal reflux disease. PPIs are widely used and can be purchased over the counter without a prescription in several countries (7, 8). Although an adequate safety profile for PPIs has been established for several years, recent studies have reported that their use may be associated with the potential risk of depression (9–12). Few studies examining the association between PPI use and depression have been conducted in developing countries (9–12). Thus, further studies are needed to investigate this issue in other populations, including in sub-Saharan African countries, in which poor mental health has been reported (13). A prevalence rate of 18% for depression was reported among adults in Ethiopia (4). A recent study reported that 62% of internally displaced persons in Sudan had depression (3). Moreover, Central Sudan, like many regions in sub-Saharan Africa, faces unique health challenges in the presence of armed conflict, including a high burden of mental health disorders (14). However, specific research assessing the association between PPIs and depression in this setting is scarce. Thus, the current study aimed to examine the association between PPIs and depression among adults in Central Sudan.

## 2 Methods

### 2.1 Study design and setting

This community-based multistage survey was conducted in Elrikieb, East Gezira, in Central Sudan from April to June 2025. Elrikieb is a large village chosen because it comprises four sub-villages, whose inhabitants resemble those of the entire Gezira State. The “Strengthening the Reporting of Observational Studies in Epidemiology (STROBE) guidelines” were strictly followed (15).

### 2.2 Study population and sampling

The study population comprised men and women aged 18 years or older residing in the selected communities. Exclusion criteria were as follows: an inability to provide informed consent, severe cognitive impairment precluding reliable questionnaire completion, and those who would not ensure the Patient Health Questionnaire-9 (PHQ-9) primarily reflects depressive symptoms.

A multistage stratified random sampling approach was employed to select participants. Within each selected sub-village, households were randomly chosen.

### 2.3 Sample size calculation

A sample size of 914 participants was calculated based on the assumption that approximately 30% of adults would have depression. This assumption was based on the findings of a previous study in Northern Sudan, which reported that 39% of adults had depression-anxiety (16). Additionally, depending on the earlier reports on drugs used in Sudan (17), we assumed that approximately 30% of depressed adults would have used PPIs. In comparison, 20% of adults without depression would have used PPIs. An analysis with this sample size would have 80% power and an alpha level of 0.05 for statistical significance.

### 2.4 Data collection

After the participants provided written informed consent, data were collected by trained medical officers (two men and two women) through face-to-face interviews using a questionnaire. The questionnaires included data on sociodemographic factors, such as age, sex, education level, marital status, employment status, smoking status, alcohol use, medical history, including diabetes mellitus and hypertension, as well as other medications used, including PPIs. In cases where patients reported a history of using PPIs, participants were asked to show the medical officer the tabs/cap to confirm their report. The participants’ weight and height were measured using the standard procedure, and their BMI was calculated as weight (kg) divided by the square of height (m^2^).

Adults were considered to have hypertension if they were diagnosed with hypertension and using medications or had newly diagnosed hypertension, which is defined by systolic blood pressure greater than or equal to 140 mm Hg or diastolic blood pressure greater than or equal to 90 mm Hg, or both. Adults were considered to have diabetes mellitus if they had already been diagnosed with diabetes mellitus.

### 2.5 Assessment of depression

The PHQ-9 was used to screen for depression. The PHQ-9 is a widely used, brief, and validated self-report tool for screening, diagnosing, and monitoring the severity of depression. Its utility in diverse cultural settings, including Arabic-speaking populations, has been established, making it a suitable tool for assessing depression in Sudan (18, 19). In this study, the Arabic validated version of the PHQ-9 consists of nine items, each scored from 0 to 3, yielding a total score ranging from 0 to 27 (20). Based on recent data, a score of ≥ 8 was used as the cut-off for clinically significant depression (21). Therefore, participants with a score of ≥ 8 were classified as having significant clinical depression necessitating further evaluation and management.

### 2.6 Data analysis

All statistical analyses were performed using SPSS for Windows (version 24.0). The Shapiro–Wilk test was used to assess the normality of the data distribution (age, BMI, and PHQ-9 scores), indicating that the data were non-normally distributed. The median (interquartile range) and number/frequency (percentage) were used to summarize the sociodemographic characteristics and PHQ-9 scores. The chi-square test was used to compare proportions between the two groups. Both univariate and multivariate analyses were performed using modified Poisson regression (depression score as a continuous variable) and binary data (with depression as a categorical variable, No/Yes) as the dependent variable and sociodemographic variables (age, education, occupation, and family history of mental illness) and PPI use as independent variables. Adults were considered to have depression if the PHQ-9 score was ≥ 8. Variables with p-values < 0.2 in the univariate analysis (Poisson and binary) were included in the multivariate analysis, adjusting for potential confounders. Propensity score matching (PSM) was used to minimize selection bias by balancing the depression and non-depression groups using the “MatchIt” package in R. The package executes the function Matchit, using depression status as the dependent variable; the other variables (excluding PPI use) are covariates, including age, marital status, BMI, sex, educational level, occupation, diabetes mellitus, hypertension, smoking status, and alcohol consumption. The test was performed using the nearest-neighbor match method at a 1:4 ratio, with a caliber width of 0.03. Adjusted odds ratios, adjusted relative risk (ARR), and 95% confidence intervals (CIs) were reported. A two-sided p-value of < 0.05 was considered to indicate statistical significance.

## 3 Results

### 3.1 General characteristics

We enrolled 914 adults (317 [34.7%] men and 597 [65.3%] women). Participants’ median (interquartile range) age and BMI were 38.0 (27.0–52.0) years and 21.6 (18.7–25.8) kg/m (2), respectively. Of the 914 participants, 238 (26.3%) had a secondary education level or higher, and 288 (31.5%) were employed. Additionally, 646 (78.2%) participants were married, 153 (16.7%) were smokers, 72 (7.9%) were alcoholics, 296 (32.4%) were hypertensive, and 78 (8.5%) had diabetes mellitus. Of the participants, 255 (27.9%) reported using PPIs (**Table 1**). Omeprazole, pantoprazole, lansoprazole, and esomeprazole were the most commonly used PPIs.

**Table 1:** General characteristics of adults in Gezira, Sudan, 2025 (n = 914).

Characteristics			Median	Interquartile range
Age, years			38.0	27.0–52.0
Body mass index, kg/m^2^			21.6	18.7–25.8
			Number	Percentage
Sex		Male	317	34.7
		Female	597	65.3
Education status		≥ Secondary	238	26.0
		< Secondary	676	74.0
Occupation status		Employed	288	31.5
		Unemployed	626	68.5
Marital status		Married	646	70.7
		Unmarried/divorced	68	29.3
Smoking status		No	761	83.3
		Yes	153	16.7
Alcohol consumption		No	842	92.1
		Yes	72	7.9
Diabetes mellitus		No	836	91.5
		Yes	78	8.5
Hypertension		No	618	67.6
		Yes	296	32.4
Proton pump inhibitor intake		No	659	72.1
		Yes	255	27.9

### 3.2 Factors associated with the depression score

**Figure 1:**
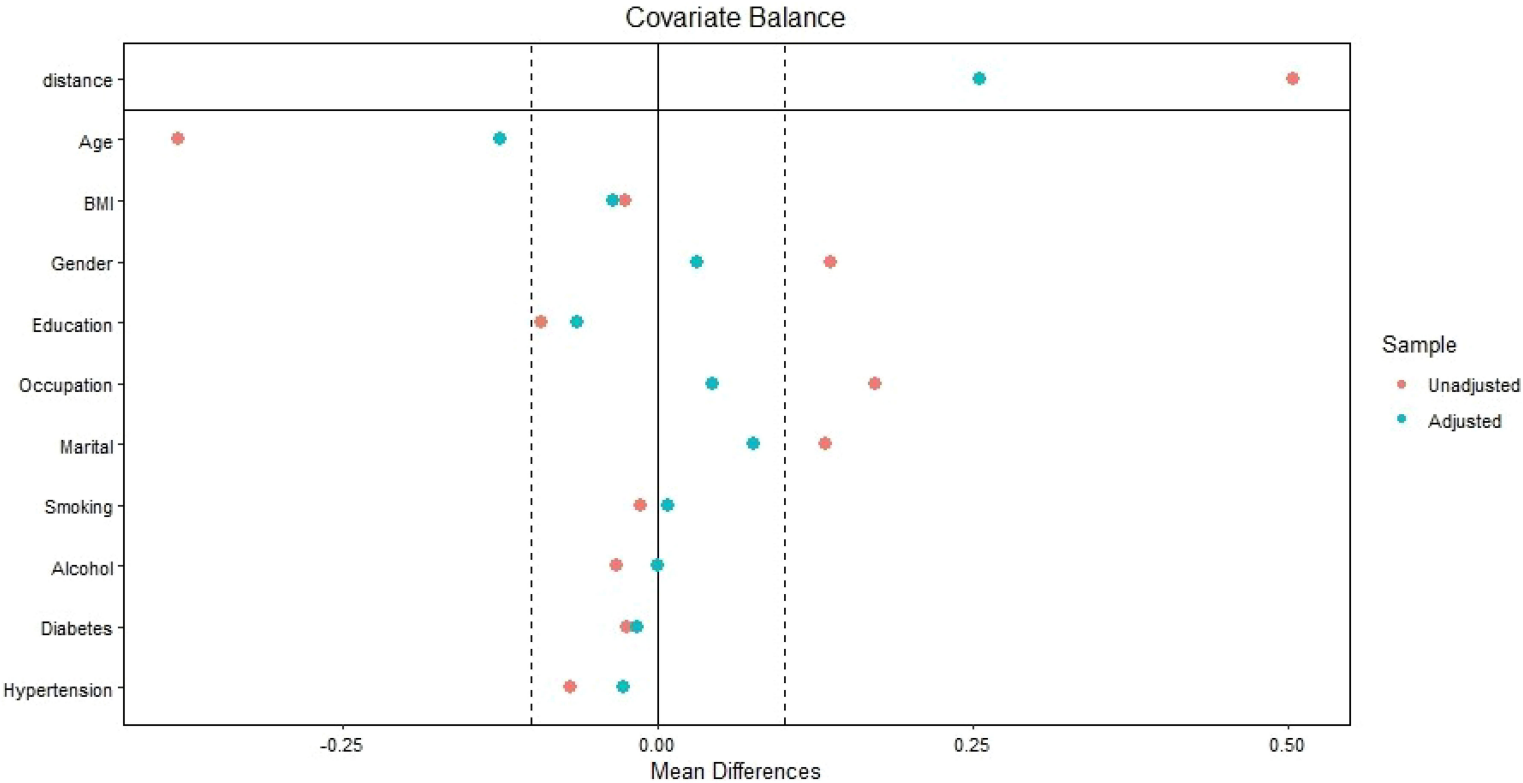
Love plot showing covariate balance between the covariates before (red points) and after (green points) propensity matching score in East Gezira, Sudan, 2025.

In univariate Poisson regression, age, being female, education, occupation, marital status, hypertension, and use of PPIs were associated with depression score. Smoking, alcohol, diabetes mellitus, and BMI were not associated with depression score. After shifting the variables to a multivariate Poisson regression, age, being female, education, marital status, and use of PPIs were associated with depression score. Thus, after adjusting for confounders, PPI use (ARR = 1.30, 95% CI = 1.21–1.40) was associated with depression score. Age (ARR = 0.99, 95% CI = 0.993–0.998) and education (ARR = 0.88, 95% CI = 0.82–0.95) were inversely associated with depression score. Being female (ARR = 1.27, 95% CI = 1.13–1.42) and being unmarried/divorced (ARR = 1.12, 95% CI = 1.04–1.20) were positively associated with depression score (**Table 2**, **Figure 1**).

**Table 2:** Univariate and multivariate modified Poisson regression analysis of factors associated with depression score among men in East Gezira, Sudan, 2025 (n = 914).

		Univariate analysis			Multivariate analysis		
Characteristics		Relative risk	95% confidence interval	P value	Adjusted relative risk	95% confidence interval	P value
Age, years		0.99	0.990–0.994	< 0.001	0.995	0.993–0.998	< 0.001
Body mass index, kg/m^2^		0.99	0.90–1.01	0.645			
Sex	Male	Reference		< 0.001			< 0.001
	Female	1.13	1.22–1.41		1.27	1.13–1.42	
Education status	≥ Secondary	Reference		< 0.001		Reference	< 0.001
	< Secondary	0.83	0.77–0.89		0.88	0.82–0.95	
Occupation status	Employed	Reference		< 0.001		Reference	0.141
	Unemployed	1.18	1.09–1.26		0.91	0.82–1.02	
Marital status	Married	Reference		< 0.001		Reference	
	Unmarried/divorced	1.18	1.10–1.27		1.12	1.04–1.20	0.002
Smoking status	No	Reference		0.830		Reference	
	Yes	1.01	0.92–1.10				
Alcohol consumption	No	Reference		0.931	Reference		
	Yes	1.01	0.89–1.12				
Proton pump inhibitor intake	No	Reference		< 0.001		Reference	
	Yes	1.32	1.23–1.41		1.30	1.21–1.40	< 0.001
Diabetes mellitus	No	Reference			Reference		
	Yes	1.11	0.99–1.26	0.069	0.92	0.81–1.04	0.210
Hypertension	No	Reference		< 0.001	Reference		0.117
	Yes	1.14	1.06–1.20		1.06	0.98–1.14	

### 3.3 Factors associated with depression

The results revealed that 155 (17.0%) participants had depression. Before matching (PSM), there was a significantly higher number of adults who used PPIs among adults with depression compared with adults without depression [57/155(36.8% versus 198/759(26.1%), P = 0.008]. Age was significantly lower in adults with depression compared with adults without depression. In univariate analysis, age, being female, being married, having a lower level of education, occupation, and using PPIs were associated with depression. BMI, alcohol use, smoking, diabetes mellitus, and hypertension were not associated with depression (**Table 3**). When variables with P < 0.20 were included in the multivariate binary regression, using PPIs was associated with depression, with an adjusted odds ratio (AOR) of 1.64 (95% CI, 1.14–2.37). Age was inversely associated with depression (AOR = 0.98, 95% CI, 0.97–0.99). Being unmarried/divorced (AOR = 1.59, 95% CI, 1.09–2.32) was associated with depression. Females, level of education, and occupation were not associated with depression in multivariate analysis (confounders), **Table 4**.

**Table 3:** Univariate binary analysis of factors (before matching) associated with depression in adults in East Gezira, Sudan, 2025 (n = 914).

		Adults with depression (n = 155)	Adults without depression (n = 795)	Univariate analysis	
Characteristics		Median (interquartile range)	Median (interquartile range)	Odds ratio (95% confidence interval)	P value
Age, years		33.0 (23.0–45.0)	40.0 (28.0–55.0)	0.97 (0.96–0.98)	< 0.001
Body mass index, kg/m^2^		21.3 (18.6–25.0)	21.9 (18.7–25.8)	0.99 (0.97–1.01)	0.786
		Number (%)	Number (%)		
Sex	Male	36(23.2)	281(37.0)	Reference	0.001
	Female	119(76.8)	478(63.1)	1.94 (1.30–2.90)	
Education status	≥ Secondary	52 (33.5)	186 (24.5)	Reference	0.020
	< Secondary	103 (66.5)	573 (75.5)	0.64 (0.44–0.93)	
Occupation status	Employed	37 (23.9)	251 (33.1)	Reference	0.026
	Unemployed	118 (76.1)	508 (66.9)	1.57 (1.05–2.34)	
Marital status	Married	92 (59.4)	554 (73.0)	Reference	0.001
	Unmarried/divorced	63 (40.6)	205 (27.0)	1.85 (1.29–2.64)	
Smoking status	No	131 (84.5)	630 (83.0)	Reference	0.464
	Yes	24 (15.5)	129 (17.0)	0.89 (0.55–1.43)	
Alcohol consumption	No	147 (94.8)	695 (91.6)	Reference	0.173
	Yes	8 (5.2)	64 (8.4)	0.59 (0.27–1.25)	
Proton pump inhibitor intake	No	98 (63.2)	561 (73.9)	Reference	0.007
	Yes	57 (36.8)	198 (26.1)	1.64 (1.14–2.37)	
Diabetes mellitus	No	145 (93.5)	691 (91.0)	Reference	0.311
	Yes	10 (6.5)	35 (9.0)	0.70 (0.35–1.39)	
Hypertension	No	114 (73.5)	504 (66.4)	Reference	0.084
	Yes	41 (26.5)	255 (33.6)	0.71 (0.48–1.04)	

**Table 4:** Multivariate binary analysis of factors (before and after matching) associated with depression among adults in Gezira State in Sudan, 2025 (n = 914).

		Before matching			After matching		
Variable		Adjusted odds ratio	95% confidence interval	P value	Adjusted odds ratio	95% confidence interval	P value
Age, years		0.98	0.97–0.99	0.014	0.99	0.98–1.01	0.301
Sex	Male	Reference		0.086			
	Female	1.78	0.92–3.43				
Education status	≥ Secondary	Reference		0.235	Reference		0.232
	< Secondary	0.78	0.52–1.17		0.78	0.52–1.16	
Occupation status	Employed	Reference		0.742			
	Unemployed	0.89	0.47–1.69				
Marital status	Married	Reference		0.015	Reference		0.095
	Unmarried/divorced	1.59	1.09–2.32		1.37	0.94–1.98	
Alcohol consumption	No	Reference		0.516			
	Yes	1.33	0.56–3.17				
Proton pump inhibitor intake	No	Reference		0.010	Reference		0.008
	Yes	1.64	1.12–2.41		1.66	1.13–2.41	
Hypertension	No	Reference		0.658			
	Yes	0.91	0.60–1.39				

The comparison between the variables before and after matching is shown in **Table 5** (**Figure 1**). After marching, when the variables with P< 0.20 were shifted to the multivariate binary analysis, using PPIs was associated with depression, with an adjusted odds ratio (AOR) of 1.66 (95% CI, 1.13–2.41), **Table 4**.

**Table 5:** Comparing factors between adults with and without depression before and after matching in East Gezira, Sudan, 2025.

		Before matching			After matching		
		Adults with depression (n = 155)	Adults without depression (n = 759)	P	Adults with depression (n = 155)	Adults without depression (n = 620)	P
Characteristics		Median (interquartile range)			Median (interquartile range)		
Age, years		33.0 (23.0–45.0)	40.0 (28.0–55.0)	<0.001	33.0 (23.0–45.0)	35.0 (26.0–45.0)	0.033
Body mass index, kg/m^2^		21.3 (18.6–25.0)	21.9 (18.7–25.8)	0.453	21.3 (18.6–25.0)	21.7 (18.6–26.1)	0.580
		Number (%)			Number (%)		
Sex	Male	36(23.2)	281(37.0)	0.001	36(23.2)	163(26.3)	0.473
	Female	119(76.8)	478(63.1)		119(76.8)	457(73.7)	
Education status	≥ Secondary	52 (33.5)	186 (24.5)	0.021	52 (33.5)	173 (27.9)	0.138
	< Secondary	103 (66.5)	573 (75.5)		103 (66.5)	447 (72.1)	
Occupation status	Employed	37 (23.9)	251 (33.1)	0.025	37 (23.9)	159 (25.6)	0.896
	Unemployed	118 (76.1)	508 (66.9)		118 (76.1)	461 (74.4)	
Marital status	Married	92 (59.4)	554 (73.0)	0.001	92 (59.4)	419 (67.6)	0.088
	Unmarried/divorced	63 (40.6)	205 (27.0)		63 (40.6)	201 (32.4)	
Smoking status	No	131 (84.5)	630 (83.0)	0.724	131 (84.5)	529 (85.3)	0.801
	Yes	24 (15.5)	129 (17.0)		24 (15.5)	91 (14.7)	
Alcohol consumption	No	147 (94.8)	695 (91.6)	0.193	147 (94.8)	588 (94.8)	0.594
	Yes	8 (5.2)	64 (8.4)		8 (5.2)	32 (5.2)	
Proton pump inhibitor intake	No	98 (63.2)	561 (73.9)	0.008	98 (63.2)	452 (72.9)	0.023
	Yes	57 (36.8)	198 (26.1)		57 (36.8)	168 (27.1)	
Diabetes mellitus	No	138 (89.0)	698 (92.0)	0.348	138 (89.0)	542 (87.4)	0.682
	Yes	17 (11.0)	61 (8.0)		17 (11.0)	78 (12.6)	
Hypertension	No	114 (73.5)	504 (66.4)	0.090	114 (73.5)	439 (70.8)	0.552
	Yes	41 (26.5)	255 (33.6)		41 (26.5)	181 (29.2)	

## 4 Discussion

The findings of the current study revealed that using PPI was associated with higher depression scores (30.0%, ARR = 1.30, 95% CI = 1.21–1.40) and a greater odds ratio of depression (1.64, 95% CI = 1.14–2.37) before and after PSM. These findings are in accord with previous research conducted in other countries (10, 11, 22–24).

The United States National Health and Nutrition Examination Survey of 16,881 adults aged 20 years or older reported that PPI use was associated with depression (22). Laudisio et al. used the Geriatric Depression Scale in older patients (n = 344) aged > 57 years in Tuscania, Italy, and found that the use of PPIs was associated with a higher odds of depression (OR = 2.38; 95% CI = 1.02–5.5) (23). Huang et al. analyzed data from the Taiwan National Health Insurance Research Database (NHIRD), revealing that the use of PPIs was associated with depression (24). In a study in Sweden of 29,320 children aged 7–17 years, Wang et al. found that the use of PPIs was associated with a higher risk of depression (10). Moreover, PPIs were examined as part of studies of a recently developed tool (magnesium depletion score) to assess magnesium deficiency, indicating an association between PPI use and depression (2, 25, 26).

Notably, in addition to an association between PPI use and depression, a previous study reported causal relationships between peptic ulcer, gastroesophageal reflux disease (which are indicators for PPI use), and depression (11). These findings should be considered when assessing the association between PPI use and depression.

However, because this study is cross-sectional, the observed relationship needs to be interpreted with caution because PPIs are typically prescribed for gastrointestinal disorders such as peptic ulcer disease and gastroesophageal reflux, and these conditions themselves were reported to contribute to depressive symptoms through pain, chronic discomfort, sleep disturbance, and reduced quality of life. In our study, we did not collect data on specific gastrointestinal disorders (symptoms, severity, and complications). Therefore, we would not be able to adjust for this potential confounding factor. This means that the observed association may partly reflect the burden of gastrointestinal disease among PPI users rather than an independent effect of PPI use. Therefore, the findings of this relationship need to be considered as hypothesis-generating and may highlight a subgroup of adults with chronic gastrointestinal symptoms who also have a higher burden of depression and may benefit from additional gastrointestinal and mental health exploration. A larger longitudinal study, such as one that administers surveys before and after PPI use, may help examine these effects in more detail.

The precise mechanism of the association between PPIs and neuropathies/depression is unknown. However, a plausible explanation is that PPIs can lead to increased serum gastrin levels, which play a role in regulating and modifying aspects of behavior that can result in depression (27, 28). Moreover, increased gastrin levels can activate cholecystokinin type B receptors in the brain, leading to depression (29). PPIs cross the blood–brain barrier, thereby interfering with neuronal function or inducing vitamin B12 deficiency. PPIs can reduce B12 absorption by inhibiting gastric acid secretion, potentially leading to B12 deficiency. B12 deficiency can lead to an increased homocysteine level, facilitating tau hyperphosphorylation and amyloid-β (Aβ) deposition, which are associated with neuropathies and depression. Likewise, it has been postulated that PPIs can inhibit vacuolar ATPases, modulate microglial Aβ phagocytosis, and neurodegeneration (9).

### 4.1 Limitations of the study

The study is limited -e.g., collecting depression data from a single baseline, inability to track exact PPI usage, and lack of reporting of comorbid gastrointestinal diseases (e.g., stress ulcer, GERD) – there is not enough evidence to establish a conclusion. PPI users are likely to have gastrointestinal diseases, which can cause stress and depression.

## 5 Conclusion

The current study showed a positive association between PPIs and depression. Longitudinal studies will be necessary to examine this relationship in more depth.

## Data Availability

The raw data supporting the conclusions of this article will be made available by the authors, without undue reservation.
